# Novel molecular imaging platform for monitoring oncological kinases

**DOI:** 10.1186/1475-2867-10-23

**Published:** 2010-07-08

**Authors:** Shyam Nyati, Brian D Ross, Alnawaz Rehemtulla, Mahaveer S Bhojani

**Affiliations:** 1 Department of Radiation Oncology, University of Michigan, Ann Arbor MI 48109 USA; 2 Department of Radiology, University of Michigan, Ann Arbor MI 48109 USA; 3 Department of Biological Chemistry, University of Michigan, Ann Arbor MI 48109 USA; 4 Center for Molecular Imaging, University of Michigan, Ann Arbor MI 48109 USA

## Abstract

Recent advances in oncology have lead to identification of a plethora of alterations in signaling pathways that are critical to oncogenesis and propagation of malignancy. Among the biomarkers identified, dysregulated kinases and associated changes in signaling cascade received the lion's share of scientific attention and have been under extensive investigations with goal of targeting them for anti-cancer therapy. Discovery of new drugs is immensely facilitated by molecular imaging technology which enables non-invasive, real time, dynamic imaging and quantification of kinase activity. Here, we review recent development of novel kinase reporters based on conformation dependent complementation of firefly luciferase to monitor kinase activity. Such reporter system provides unique insights into the pharmacokinetics and pharmacodynamics of drugs that modulate kinase signaling and have a huge potential in drug discovery, validation, and drug-target interactions.

## Introduction

Cell signaling is a mode of communication by which the intracellular information is conveyed from the site of instigation to the site of action. Recent advances in molecular profiling technologies such as microarrays and proteomics along with synergistic growth in the field of bio-informatics, have actuated our appreciation of signaling changes in patho-physiological conditions and led to identification of unique disease biomarkers [1-5]. For example growth factor such as EGFR or Her-2, may be considered as biomarkers in certain human cancers where they are amplified, overexpressed and/or mutated and immensely alter the downstream signaling [6-12]. Identification of such unique central regulators in the disease signaling has lead to development of targeted molecular drugs [6,12]. Although, number of these disease biomarkers have been identified and characterized, the true impact of these understandings will be felt only when applied to diagnosis, staging and treatment of patients. Currently, these innovative developments in understanding the role of biomarkers in human malignancy have minimally ameliorated clinical oncology. This is partly due to the fact that most of the efforts are focused on identifying biomarkers from cancer samples obtained by biopsy of tumors which provide a frozen snapshot of biomarkers at the time of sample retrieval and fail to provide any information on the dynamic changes within the malignancy and its milieu [13]. Therefore, concurrent innovations are needed for real time and non-invasive monitoring of biomarker and events they modulate in live cells or organisms [14].

Molecular imaging is a recent area of investigation that attempt to develop suitable probes for noninvasive visual representation of biological processes at the cellular and molecular level in the whole organism and the modalities and instrumentation to support the visualization and quantification of these processes. This is an attempt to bridge the gap between discovery of biomarkers and their deployment in clinic. At present molecular imaging is still largely in the animal experimental phase but promises to bring dramatic change in the way in which a disease is diagnosed, staged and treated. In clinical oncology it will allow oncologists to diagnose cancer at an earlier stage based on molecular characterization, predict the risk of precancerous lesion progression, quantify activities of specific molecules related to tumor growth, invasion and metastasis, select a rational molecular therapy and assess the efficacy of chemo and radio therapeutic agents in real time [15-21]. Over the past several years three different noninvasive imaging technologies have been fine tuned for prime time: (A) optical imaging (bioluminescence and fluorescence imaging) [22-24]; (B) magnetic resonance imaging [MRI][25]; (C) nuclear imaging (e.g single photon emission computed tomography [SPECT] and positron emission tomography [PET]) [26-28]. These have been extensively discussed in a number of reviews and book chapters [13,14,29-32]. In this article, we will discuss the recent development in the field of bioluminescent optical imaging for monitoring signaling cascades with special emphasis on luciferase complementation platforms for imaging of kinases.

## Bioluminescent optical reporters and complementation assays

Discovery of reporters that are genetically encoded and generate light such as fluorescent proteins and luciferases in conjunction with the development of instrumentation for real time functional imaging of their activity has offered researchers powerful tools to perform noninvasive studies of dynamic biological process in intact cells and whole organisms. These optical reporter systems have extensively utilized in molecular imaging of signaling pathways mainly because of their efficiency for sequential imaging, operational simplicity, and substantial cost benefits. Bioluminescent firefly luciferase based reporters are widely used for non-invasive, real-time, repetitive imaging both *in vitro *and *in vivo*. For monitoring signaling cascade or activity of specific biomarker *in vivo*, firefly luciferase is the reporter of choice as 30% of the light generated by firefly luciferase has an emission spectra above 600 nm, a region where the signal attenuation by the absorbing and scattering properties of live mammalian tissue is minimal [33,34]. A major disadvantage of luciferases, like other genetically encoded reporters, is that their use in clinical setting is contingent upon the acceptance of gene therapy protocols for patients. However, in basic research they exhibit a principal advantage in assessing a variety of biological functions including transcriptional and translational regulation, signal transduction, protein-protein interaction, oncogenic and viral transformations, cell migration and trafficking and monitoring tumor burden [35-41] Additionally, a number of modifications have been described for targeting/expressing these reporters in specific organelle, cells or organs by exploiting inducible promoters and regulatory elements [42-46].

**Figure 1:**
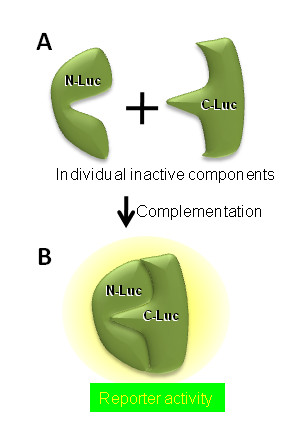
**Principle of luciferase complementation based reporter**. When luciferase is split to two components (A), each of the units is incapable of generating bioluminescence. However, when the individual components are brought in close proximity, the luciferase enzymatic activity is restored because of intramolecular complementation (B).

Protein complementation assay have garnered a lot of lime light for monitoring protein-protein interaction, kinase and protease activities [47]. Here, the monomeric reporter is split into two separate inactive components in such a way that when these components are brought into close proximity they re-constitute the original reporter activity (Figure 1). Complementation for a number of reporters have been developed for understanding mammalian biology. These include fluorescent proteins (GFP and YFP), bioluminescent enzymes (Firefly Luciferase, Renilla Luciferase, Gaussia Luciferase; Figure 1), β-galactosidase, dihydrofolate reductase (DHFR) and TME1 b-lactamase [48-54]. Luker et al. optimized firefly luciferase protein complementation by screening incremental truncation libraries of N- and C-terminal fragments of luciferase [50]. They utilized the complementation assay for demonstrating the phosphorylation dependent interaction between human Cdc25C and 14-3-3-e *in vitro *and FRB-FKBP12 interaction *in vivo *in real time non-invasively. On similar lines Paulmurugan and Gambhir [55] developed Renilla luciferase complementation assay and monitored in real time the interaction of MyoD and Id. Similarly, Gaussia luciferase complementation assay were developed by Remy and Michnick [53] where they monitored crosstalk of TGFb and insulin signaling. Li et al [56] reported development of luciferase complementation based probes for ligand dependent EGFR dimerization and activation. We have utilized the firefly luciferase complementation for monitoring Akt kinase and caspase-3 protease activity [30,57-59].

## Imaging of kinases

Protein kinases are one of the principle regulators of signaling cascades influencing majority of cellular decisions [60-65]. Protein kinases posttranslationally modify a substrate protein by covalent attachment of a phosphate group to a specific amino acid. This phosphorylation of substrate proteins can be mediated by protein Ser/Thr kinases (at serine or threonine residue) or by protein Tyr kinases (at tyrosine residue). Phosphorylation of target residues in proteins results in changes in substrate activity, sub-cellular location or/and interaction with other proteins [30]. These changes mediate a bulk of signaling in normal eukaryotic cells and are very tightly controlled by autoinhibitory and regulatory constraints which act as a safeguard for aberrant kinase activation [62-66]. Dysregulation and mutations in kinase activity have been reported to play a causal role in more than 400 human diseases including as cancer, neurological disorders, rheumatoid arthritis, and psoriasis [66-71]. Although we have met with colossal success in identification of aberrant kinases in a plethora of diseases, the translation of this information to clinic has been much less successful such that majority of these biomarkers remain undrugged. Therefore, fresh impetus is needed in the areas that will allow identification of novel inhibitors for kinase biomarkers. Towards this, we need the molecular imaging modalities that have the potential to be adapted for high through put screening of inhibitor libraries.

**Figure 2:**
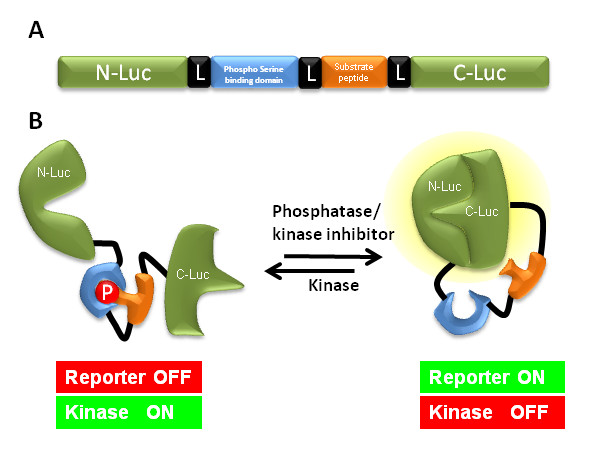
**The domain structure and mechanism of action of a kinase reporter**. (**A) **N-Luc (amino acids 2-416) and C-Luc (amino acids 398-550) are the amino- and carboxy-terminal domains of firefly luciferase that are fused to the appropriate ends of the reporter. The peptide domain constitutes a kinase substrate sequence with a flexible linker (L) containing of GlyGlySerGlyGly on either side. Yeast Rad52p FHA2 phospho-Ser/Thr binding domain (residues 420-582) attached to the amino-terminal of substrate peptide domain. (**B**) The proposed mechanism of action for the split luciferase based kinase reporter involves kinase dependent phosphorylation of the target peptide which results in its interaction with the FHA2 domain. In this form the reporter has minimal bioluminescence activity. In the absence of kinase activity, association of the N-Luc and C-Luc domains restores bioluminescence activity.

**Figure 3:**
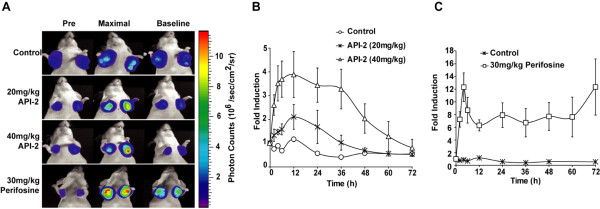
**Imaging of pharmacodynamics of PI3K/AKT-kinase inhibitors in live animals **[57]. **(A) **Mice transplanted with D54 cells stably expressing bioluminescent Akt reporter (BAR) were treated with vehicle control (20% DMSO in PBS), API-2 (20 mg/kg or 40 mg/kg) or perifosine (30 mg/kg). Images of representative mice are shown before treatment, during maximal luciferase signal upon treatment (Max), and after treatment. **(B) **Tumor-specific bioluminescence activity of D54 cells stably expressing BAR, treated with either the vehicle control (20% DMSO in PBS) or API-2 (20 mg/kg or 40 mg/kg), was monitored at various times. Fold induction of signal intensity over pretreatment values was plotted as mean ± s.e.m. for each of the groups. **(C) **Bioluminescence activity in tumor-bearing mice before treatment and in response to treatment with 30 mg/kg perifosine, plotted as fold induction over pretreatment values (± s.e.m.) for each of the groups.

We have recently developed a luciferase complementation based kinase imaging platform that allows quantitative, real time, non-invasive imaging of kinase activity (Figure 1) and is easily amenable for high throughput screening of new drugs [30,57,58]. We have utilized this technology to monitor Akt, one of the best characterized serine threonine kinases that is involved in tumor initiation, progression and resistance to cancer treatment and is a central signaling hub wherein many upstream oncogenic stimuli such as growth factor signaling and cytokine cascades converge [72]. This recombinant bioluminescent Akt reporter, (BAR) was constructed by fusion of an Akt consensus substrate peptide and phospho-amino acid binding domain (FHA2) which were flanked by the amino- (N-Luc) and carboxyl- (C-Luc) terminal domains of the firefly luciferase reporter molecule (figure 2). In the presence of Akt kinase activity, phosphorylation of the Akt consensus substrate sequences within the reporter results in its interaction with the FHA2 domain, thus stearically preventing reconstitution of a functional luciferase reporter molecule. In the absence of Akt kinase activity, release of this stearic constraint allows reconstitution of the luciferase reporter molecule whose activity can be detected non-invasively by bioluminescent imaging (BLI). The inhibition of Akt activity using an Akt inhibitor, API2 and a PI-3K inhibitor, perifosine resulted in an increase of bioluminescence activity in a time- and dose-dependent manner (figure 3), which indicated that BAR provides a surrogate for Akt activity in terms of quantity and dynamics [57,58]. BAR was also used to study upstream signaling events of Akt. For example, stimulation of EGFR could be evaluated using Akt activity as a surrogate and monitored by bioluminescent imaging [57]. The use of an EGFR inhibitor, erlotinib in the erlotinib-sensitive and -resistant cell lines resulted in differential activation of the BAR reporter. In summary, BAR allows imaging of signaling leading to activation/inactivation of Akt in a quantitative, dynamic and non-invasive manner and that this kinase imaging platform may be adapted for other kinases.

*In vivo *imaging in animals is a significant advantage of bioluminescent kinase which allows for an enhanced understanding of pharmacokinetics and bioavailability of specific drugs. For example, at 40 mg/kg API-2 treatment, peak inhibition was detected at 12 hours and inhibitory levels of the compound were detected for up to 24 hours (high bioluminescence) but decreased thereafter (figure 3B). In contrast, when 20 mg/kg was delivered, although peak inhibition was detected at 12 hours, a decrease in reporter activity was measured in subsequent measurements [57]. Unlike API-2 for which published pharmacokinetics data are not available, the pharmacokinetics of perifosine has been extensively studied. Published data demonstrated that high plasma concentrations of the drug could be detected for as long as seven days post treatment [73,74]. The high levels of Akt inhibitory activity for three days detected with non-invasive BAR supports the above observation (figure 3C). Further, Perifosine induced a 12 fold induction in BAR bioluminescence activity while API-2 showed only 4-fold. This difference may be reflective of their bioavailability at the tumor site. Such studies establish an important role for bioluminescent kinase imaging platform in detection of *in vivo *drug-target interaction.

A major utility of bioluminescent kinase reporter is for high throughput screening of a library of inhibitors. Such cell based assays provide a major advantage in that only compounds that interact with the target in the correct cellular compartment and under normal cellular physiological conditions of that compartment (pH, concentrations of specific ions etc.) would be identified. In contrast to other cell based reporter screens, which are fraught with false positives, the kinase reporter described here is a "gain of function assay" wherein the inhibition of kinase activity results in an increase in bioluminescence. For example, compounds that kill cells (and thus result in a loss of signal) or those that inhibit luciferase activity may show up as false positives in typical luciferase/fluorescent/enzyme based assays. However, specific inhibition of reporters like BAR result in an increase in bioluminescence activity and thus non-specific cytotoxic agents are eliminated. Such carefully designed screening methodology will enable us to narrow down the number of positive compounds to a smaller group of "true positives".

Optimum activity and specificity of a kinase is dependent upon its subcellular localization. For example, Akt is recruited to the plasma membrane by PI-3 kinase-generated D3-phosphorylated phosphoinositides which bind to the Akt PH domain and induce the translocation [75,76]. At the cell membrane, phosphoinositide-dependent kinase-1, co-localizes and phosphorylates within the activation loop of Akt [75,76]. Therefore, a membrane targeted reporter for Akt activity is more likely to have higher activity. Indeed, the sensitivity of MyrPalm-BAR reporter was more than twice as much when compared with BAR alone in reporting Akt signaling [30]. Thus, utilization of subcellular information of kinases may optimize the kinase reporters and therefore must be employed for reporter construction.

As discussed above, the BAR can be adapted for other protein kinases including receptor or non-receptor Tyr and Ser/Thr kinases by using a suitable substrate and a specific phospho-amino acid binding protein domain. We have successfully adapted this platform for monitoring GSK3β/CK1α kinase activities using a β-catenin substrate sequence [77]. Towards a comprehensive understanding of oncological signaling and accelerating the drug discovery process, we are currently developing imaging tools for several high priority oncological targets such as EGFR, Her2, c-Met, Ras-Raf-MEK-ERK (MAPK), mTor, and TGFβ receptor. Further, to aid in identifying the target phosphorylation site, there are a number of resources and methodologies described in literature [78-88] and on the web such as http://www.kinasenet.ca, http://www.phosphosite.org, http://www.kinase.com. Experimentally verified phosphorylation sites are available at http://phospho.elm.eu.org and for prediction of phosphorylation site http://www.cbs.dtu.dk/services/NetPhos/ or http://scansite.mit.edu may be useful.

In summary, molecular imaging reporters for kinases provide a unique opportunity to monitor cellular pathways both *in vitro *and *in vivo*. This greatly facilitates the real time visualization of the aberrant oncological signaling and will play an important role in monitoring therapeutic outcome, drug-target validation as well as identification of next generation of drugs.

## Competing interests

The authors declare that they have no competing interests.

## Author's contributions

SN and MSB wrote the review and made the figures. AR and BDR revised the manuscript.
